# A comparison of knowledge and practices of universal precautions among public sector health care workers in Ugu north sub-district, KwaZulu-Natal, South Africa (2010–2014)

**DOI:** 10.4102/sajid.v35i1.162

**Published:** 2020-09-04

**Authors:** Renee Govender, Saloshni Naidoo

**Affiliations:** 1 Ugu Health District, KwaZulu-Natal Provincial Department of Health, KwaZulu-Natal, Port Shepstone, South Africa; 2 Discipline of Public Health Medicine, School of Nursing and Public Health, University of KwaZulu-Natal, Durban, South Africa

**Keywords:** universal precautions, health care workers, needlestick injuries, public health, infection control

## Abstract

**Background:**

Annually, there are a high number of needlestick injuries (NSIs) among health care workers (HCWs) globally. The knowledge and practice of HCWs of universal precautions (UPs) play an important role in determining the risk of an NSI. The objective of this study was to compare the knowledge and practices of UPs among HCWs with NSIs with HCWs without NSIs, in Ugu north sub-district in KwaZulu-Natal (KZN), South Africa, between 2010 and 2014.

**Methods:**

A study among HCWs having an NSI (*n* = 100) between 2010 and 2014 compared with controls (*n* = 200) was conducted in 2016–2017 at a district hospital and 11 primary health care facilities in Ugu north sub-district, KZN, South Africa. Health care workers’ knowledge and practices of UPs were assessed by using a standardised questionnaire. Knowledge and practice responses were scored, and means and standard deviations (SDs) were calculated. Total scores of knowledge and practices were categorised into acceptable and unacceptable, and a binary logistic model was used to identify independent factors associated with being a case. The accepted level of significance was 0.05.

**Results:**

The majority of the participants were nurses (*n* = 233; 77.7%) and female (*n* = 227; 75.7%). Control HCWs had better practice scores for UPs (86.13%; SD: 16.57) compared with cases (82.43%; SD: 19.98). The logistic regression analysis showed that the HCWs with acceptable knowledge and unacceptable practice were more likely to have had an NSI (odds ratio [OR]: 5.8; 95% confidence interval [CI]: 1.4–24.0).

**Conclusion:**

There were significant differences between cases and controls with respect to knowledge and practice of UPs that are important findings for workplace health and safety and HCW training.

## Introduction

Needlestick injury (NSI) is one of the most common routes of transmission of occupationally acquired blood-borne diseases in health care workers (HCWs). A systematic review by Cooke and Stephens in 2017 found that between 14.9% and 69.4% of HCWs globally reported having an NSI, and that NSIs were responsible for 37% – 39% of the global burden of hepatitis B and C infections in HCWs.^1^

The World Health Organization (WHO)^2^ and the Centers for Disease Control^3^ recommend the practice of universal precautions (UPs) by HCWs as a means of preventing and reducing the transmission of blood-borne diseases in health care settings. The elements of UPs include hand hygiene (washing and drying), respiratory hygiene or cough etiquette and use of personal protective equipment such as gloves, masks, coats, closed shoes, patient care equipment (protective clothing), care of the environment, clean textiles and laundry, safe injection practices (safe handling and disposal of sharps) and isolation precautions.^2,3^ Compliance with UPs has minimised the risk of health care infections and reduced NSIs.^4,5,6^ A systematic review of the efficacy of interventions to promote hand hygiene in hospitals found that there were reductions in the transmission of hospital pathogens in the presence of improved hand hygiene in the 19 studies that reported on clinical outcomes.^5^ Similarly, Yang et al. reported, in their systematic review of the impact of educational training and safeguard interventions to reduce NSIs, that double-gloving led to a reduction in NSIs among HCWs.^6^

Despite the evidence of UPs reducing the burden of blood-borne diseases, research suggests that HCWs have varying levels of knowledge and may implement poor practices with respect to UPs in health care settings.^7,8,9,10^ A systematic review conducted by Nasiri et al. found that nurses had knowledge and practice levels with respect to UPs, ranging from 21% to 90% and 37.3% to 84.3%, respectively.^8^ In a study among nurses in Iran, 22% had good knowledge and 34% reported good practice of UPs,^11^ while a study of nurses’ knowledge and practice of UPs in Delta State, Nigeria, found knowledge levels above 90% and practice scores below 50%.^12^

In South Africa, NSIs among HCWs have been regularly reported,^13,14,15^ with the potential risk for blood-borne diseases being high in this occupational group. Precautions against NSIs and the transmission of blood-borne diseases in the health care setting in South Africa are guided by the National Infection Prevention and Control Policy and Strategy^16^ and the Hazardous Biological Agents Regulation No. 1390^17^ of the *Occupational Health and Safety Act* No. 85 of 1993.^18^ Both guiding documents recommend the practice of UPs in preventing transmission of blood-borne disease in the health care setting and ensuring the training of HCWs on the content and implementation of UPs.

Despite active training initiatives within health care settings and existing guidelines, knowledge and practice of UPs among HCWs in South Africa appear to mirror that of the rest of the world.^19,20^ Massinga et al. reported on the limited knowledge and negative attitudes, which influenced the practice of UPs among nurses working in operating theatres in hospitals in northern KwaZulu-Natal (KZN).^19^ Nieuwoudt in an intervention study among nurses in the Cape Winelands and Overberg District found that although cases and controls had good knowledge of UPs (100%), they had poor adherence with respect to elements such as sharp management where more than 98% of cases and controls reported reusing needles for antibiotic infusions.^20^ Knowledge and practice of UPs are important in preventing NSIs in the health care setting. There is a need to understand HCWs’ knowledge and practice behaviour with respect to UPs in the South African setting and its relationship with NSIs. Thus, the aim of this study was to compare the knowledge and practices of UPs between HCWs with NSIs and those without NSIs, in the Ugu north sub-district, KZN, between 2010 and 2014.

## Methods

### Study design and setting

This case–control study was conducted among the health care facilities (HCFs) in the Ugu north sub-district (Umdoni) in Ugu Health District, KZN. This sub-district located on the south coast of KZN comprises one district hospital, one emergency medical rescue services base and 11 primary health care (PHC) facilities. The hospital and two of the 11 PHC facilities operate for 24 h, and therefore the HCWs work both night and day shifts.

### Study population

The study population comprised all HCWs employed in HCFs in the Ugu north sub-district who had direct contact with patients and who had come into contact with blood-borne pathogens (BBPs). The HCWs of concern in this study were medical officers, all dental staff, all nursing categories, emergency medical and rescue services (EMRS) and general cleaners. The total staffing in the Ugu north sub-district was 866 HCWs at the time of this study, of whom 489 met the inclusion criteria and were considered for the study. Potential participants were interviewed by using a screening questionnaire that indicated whether they could be selected as cases or controls, or excluded from the study. Cases for this study were defined as all those HCWs who had had an NSI while on duty at the Ugu north sub-district HCFs, during January 2010–December 2014. Controls for this study were defined as all those HCWs who had not had an NSI while on duty at the Ugu north sub-district HCFs during January 2010–December 2014.

### Sample size

The minimum sample size required for this study was calculated based on the presence of two controls to one case. At a level of significance of 5% and power of 80%, anticipating the proportion of controls and cases to have knowledge about UPs to be 15% and 30%, respectively, a minimum of 91 cases and 182 controls were required for the study. Because of the small employee population in the sub-district, all 489 HCWs were considered for inclusion in the study and invited to participate. However, only 300 HCWs (100 cases and 200 controls) gave consent to participate in the study. Those that declined to participate indicated that their workload did not allow them time to participate, and some failed to give a reason.

### Data tools and collection

A self-administered anonymised questionnaire was used to collect data in this study. The questionnaire consisted of four sections comprising 49 closed-ended questions with the exception of age and years of employment, which were collected as continuous variables.

The first section of the questionnaire covered four questions on demographic details (age, sex, number of years employed as an HCW and work area in the facility). The second section of the questionnaire comprised 11 questions, which focussed on HCW knowledge of UPs and post-exposure prophylaxis (PEP). There were six questions on the concept of UP, potential occupational exposure paths, frequency of hand washing, diseases requiring UPs, management of body fluids requiring UPs and patient-related situations requiring UPs. The questions had multiple categorical response options with more than one option being correct. There were a further four categorical questions on PEP.

The third section of the questionnaire comprised 19 questions on the practice of UPs and factors that influenced compliance with UPs. There were five questions related to hand washing, use of gloves, mask and eye protection, gown and waterproof dressing for abrasions, with responses on a scale of ‘always’ to ‘never’. There were two questions on needle and sharps management; these had multiple response options with one response being correct.

The researcher made appointments with the manager of the hospital as well as the operational managers (OMs) of each HCF to introduce and explain the study to them. Appointments were made to visit each HCF and collect the data. On the day of visiting the PHC facilities, the research team made their presence known to the sister in charge of each facility. Self-administered questionnaires were given to those HCWs who met the inclusion criteria as a case or a control after being informed about the study and written consent was obtained. The research team was available in person to address queries that participants may have had. Participants answered and completed the questionnaire, which took approximately 30 min to complete, in their work cubicles or in the tea room with privacy being ensured at all times. The research team visited the HCFs during the day and met those HCWs going off night shift in the morning when they finished work. Facilities were revisited to ensure that all staff who were on leave were given an opportunity to participate. The researcher tested the validity of the questionnaire by conducting a pilot study and made the necessary adjustments.

### Data analysis

The data obtained from the participants’ responses to the questionnaire were re-categorised for analysis. There were 10 staff categories, which were re-classified into four categories (medical officer and dental staff, nursing staff, general cleaner and paramedic staff).

In the second section of the questionnaire, which tested the knowledge of UPs and PEP, each correct answer scored one point and each incorrect answer scored zero. Each sub-section was totalled and the total knowledge percentage scores were calculated for each sub-section, and the total overall knowledge score was calculated for each participant. Means and standard deviations (SDs) for sub-section knowledge and overall knowledge scores were calculated.

In the third section of the questionnaire, which tested UP practices, a score of one was given for each correct practice and zero for incorrect practices. Sub-section and total scores for UPs were calculated for each participant, and means and SD for sub-section and overall UP scores were calculated.

Frequencies and means and SDs were calculated for categorical data and continuous data, respectively. In bivariate analysis, the independent samples *t*-test was used to test for significant associations between cases and controls, with respect to their knowledge and practice scores. Logistic regression (unadjusted and adjusted) was used to test for associations between demographic factors, knowledge and practice of UPs, and being a case. The dependent variable investigated in this study was that the HCW reported having had an NSI. The demographic variables included in the model were age, sex, years of employment and staff category. Hepatitis vaccination history, although considered in the unadjusted model, could not be included on the adjusted model because of the small numbers of participants who had not been vaccinated.

The total scores of knowledge and practice were categorised as acceptable or unacceptable, with a score of 80% and above being considered as having acceptable knowledge and practice of UPs. Four variables were considered for inclusion in the model for knowledge and practice of UP (unacceptable knowledge and practice of UPs, acceptable knowledge and unacceptable practice, unacceptable knowledge and acceptable practice, and acceptable knowledge and practice). The accepted level of significance was 0.05 (*p* = 0.05).

The link test was used to test the model specification. The Hosmer–Lemeshow goodness-of-fit statistic was used to test the model fit. The residuals and squared residuals were also examined to see where the observations fell and to determine if there were any extreme observations. The last test that was performed was the linearity test to know the linearity of the model.

### Ethical consideration

The study was approved by the University of KwaZulu-Natal’s Biomedical Research and Ethics Committee (BREC) (reference number: BE442/15). Permission to conduct this study was given by the KwaZulu-Natal Provincial Department of Health (HRKM91/16, KZ_2016 RP14_603) and the managers of all the participating facilities. Each participant provided informed written consent to participate in the study.

## Results

### Demographic characteristics of health care workers

Three hundred (*n* = 300) participants were included in this study, with the majority of participants being nurses (*n* = 233; 77.7%), which skewed the target population of HCWs. The HCFs are run by nurses and therefore nurses make up the majority of the population. Most participants were female (cases: *n* = 80; 80%; controls: *n* = 147; 73.5%). The median age for cases was 37 years (range 23–62), whereas for the controls, it was 40 years (range 22–63). The median duration of employment was 10 years for cases (range 2–35) and 12 years for controls (range 2–40). Significantly more paramedic staff were in the control group, compared with medical officers (Table 1).

**TABLE 1:** Demographic characteristics of health care workers (cases and controls) surveyed for knowledge and practice of universal precautions (*N* = 300).

Variable	Case (*n* = 100)				Control (*n* = 200)				*p*
	*n*	%	Median	Range	*n*	%	Median	Range	
Age (years)	-	-	37	23–62	-	-	40	22–63	0.35
**Sex**	-	-	-	-	-	-	-	-	0.2
Female	80	80	-	-	147	73.5	-	-	-
Male	20	20	-	-	53	26.5	-	-	-
**Work experience**	-	-	-	-	-	-	-	-	0.15
Years of work experience as a health care worker (years)	0.7	0.7	10	2–35	0.7	0.7	12	2–40	-
**Job description (*n*)**	0.7	0.7	-	-	0.7	0.7	-	-	0.04
Medical officer or dental worker	13	13	-	-	11	5.5	-	-	-
Nursing staff	77	77	-	-	156	78	-	-	-
General cleaner	8	8	-	-	16	8	-	-	-
Paramedics	2	2	-	-	17	8.5	-	-	-
**Hepatitis B vaccination (*n*)**	-	-	-	-	-	-	-	-	0.7
Yes	99	99	-	-	196	98	-	-	-

### Comparison of knowledge about universal precautions and post-exposure prophylaxis among health care workers

Most of the cases (*n* = 98; 98%) and controls (*n* = 190; 95%) indicated that they knew what UP guidelines are (not shown in Table 1). There were significant differences in the total mean knowledge scores about UPs between cases (87.92%; SD: 13.79) and controls (83.59%; SD: 13.79) (*p* = 0.001), with cases scoring higher than controls. Knowledge about the concept of what constituted UP practices showed a significant difference between the cases (81.1%; SD: 24.34) and controls (73.9%; SD: 26.91) (*p* = 0.02).

Twenty-six per cent (*n* = 26) of cases and 20% (*n* = 40) of controls believed that recapping needles is part of the concept of UP practice (not shown in table), but when testing the knowledge of the HCWs, 18% (*n* = 18) of cases and 29.5% (*n* = 59) of controls believed that needles should be recapped after use (Table 2).

**TABLE 2:** Comparison of knowledge scores about universal precautions and post-exposure prophylaxis among health care workers (cases and controls) (*N* = 300).

Variable	Case (*n* = 100)		Control (*n* = 200)		*p*
	Mean (%)	SD	Mean (%)	SD	
Concept of universal precautions	81.1	24.35	73.9	26.92	0.02
Potential occupational exposure paths for occupational infections	60.80	28.13	52.10	26.29	0.01
Frequency of hand washing	85.50	28.47	75.50	32.87	0.01
Diseases requiring the practice of universal precautions	99.00	7.88	98.63	8.39	0.61
Management of body fluids requiring universal precautions	99.80	2.00	98.20	10.06	0.20
Patient-related situations requiring the use of universal precautions	87.00	31.08	89.60	24.08	0.92
Recapping of needles	82.00	38.60	70.50	45.72	0.03
Post-exposure prophylaxis given to HIV-negative HCWs only	87.00	-	85.00	-	0.64
Time frame for PEP administration (first dose)	99.00	10.00	97.50	15.65	0.38
Duration of PEP administration	98.00	14.07	95.00	21.85	0.21

**Total score**	**87.92**	**13.79**	**83.59**	**13.79**	**0.001**

PEP, post-exposure prophylaxis; SD, standard deviations; HCWs, health care workers; HIV, human immunodeficiency virus.

There were significant differences in knowledge scores about exposure paths for the transmission of occupational infections between cases (60.8%; SD: 28.13) and controls (52.1%; SD: 26.29) (*p* = 0.01). There was also a significant difference between cases (85.5%; SD: 28.47) and controls (75.5%; SD: 32.87) with respect to knowledge about when hand washing should be performed in terms of UPs (*p* = 0.01). Health care workers had acceptable knowledge about the situations requiring the use of UPs. Health care workers’ knowledge regarding PEP guidelines and management was acceptable with no significant difference between cases and controls (Table 2).

### Comparison of universal precaution practices among health care workers

Although there was no significant difference when comparing the total mean scores for the practices of UPs, controls (86.13%; SD: 16.57) had better scores than cases (82.43%; SD: 19.98) (*p* = 0.07). The use of gloves was found to be significantly different between the cases (92%; SD: 0.32) and controls (97.5%; SD: 0.15), respectively (*p* = 0.03). The use of masks and eye protection was also found to differ significantly between cases (69%; SD: 0.66) and controls (82.5%; SD: 0.51) (*p* = 0.01) (Table 3).

**TABLE 3:** Comparison of universal precautions practice scores among health care workers (cases and controls) (*N* = 300).

Variable	Case (*n* = 100)		Control (*n* = 200)		*p*
	Mean (%)	SD	Mean (%)	SD	
Wash hands after patient contact	81.00	0.48	88.00	0.40	0.10
Correct needle and syringe management after giving injections	74.00	1.05	74.50	1.13	0.90
Correct ‘sharps’ and needle disposal	85.00	1.07	83.00	1.12	0.70
Use of gloves	92.00	0.32	97.50	0.15	0.03
Use of gown	91.00	0.46	92.00	0.42	0.75
Use of mask and eye protection	69.00	0.66	82.50	0.51	0.01
Use of waterproof dressing for cuts and abrasions	85.00	0.48	85.50	0.49	0.90

**Total score**	**82.43**	**19.98**	**86.13**	**16.57**	**0.07**

SD, standard deviations.

### Association of demographics, knowledge of universal precaution guidelines, practices of universal precautions and exposure to needlestick injuries

The logistic regression model found that the ‘knowledge and practice’ score was the only factor significant in both the unadjusted and adjusted models. Health care workers with acceptable knowledge and unacceptable practice were more likely to have an NSI, compared with those with unacceptable knowledge and practice (odds ratio [OR]: 5.8, confidence interval [CI]: 1.4–24.0) (Table 4). The overall likelihood ratio test (Wald statistic) was significant (*p* = 0.03), indicating that the model was significant.

**TABLE 4:** Logistic regression for risks of needlestick injuries among health care workers (*N* = 300).

Variable	Case (*n* = 100)		Control (*n* = 200)		Total	Unadjusted			Adjusted		
	*n*	%	*n*	%		OR	95% CI	*p*	OR	95% CI	*p*
**Age**											
20–29 years	16	40.0	24	60.0	40	ref	-	-	ref	-	-
30–39 years	45	37.8	74	62.2	119	0.9	0.4–1.9	0.8	0.9	0.3–2.4	0.8
40–49 years	24	31.2	53	68.8	77	0.7	0.3–1.5	0.3	0.8	0.3–2.7	0.8
50–69 years	15	23.4	49	76.6	64	0.5	0.2–1.1	0.08	0.6	0.2–2.2	0.4
**Sex**											
Male	20	27.4	53	72.6	73	ref	-	-	ref	-	-
Female	80	35.2	147	64.8	227	1.4	0.8–2.6	0.2	1.5	0.8–2.8	0.2
**Years employed**											
2–5 years	18	35.3	33	64.7	51	ref	-	-	ref	-	-
6–10 years	36	40.0	54	60.0	90	1.2	0.6–2.5	0.6	1.6	0.6–4.0	0.3
11–15 years	23	31.5	50	68.5	73	0.8	0.4–1.8	0.7	1.0	0.4–2.8	0.9
16–20 years	12	27.3	32	72.7	44	0.7	0.3–1.7	0.4	1.0	0.3–3.5	0.9
21–40 years	11	26.2	31	73.8	42	0.7	0.3–1.6	0.3	1.0	0.3–3.9	0.9
**Job description**											
Medical officers or dental staff	13	54.2	11	45.8	24	ref	-	-	ref	-	-
Nurse	77	33.0	156	67.0	233	0.4	0.2–0.9	0.04	0.41	0.2–1.0	0.06
General cleaners and paramedics	10	23.3	33	76.7	43	0.3	0.09–0.7	0.01	0.29	0.1–1.0	0.04
**Hepatitis vaccination**											
Yes	99	33.6	196	66.4	295	ref	-	-	excluded	excluded	excluded
No	1	20.0	4	80.0	5	0.5	0.05-4.5	0.5	-	-	-
**Knowledge and practice**											
Knowledge and practice of UPs both unacceptable	3	16.7	15	83.3	18	ref	-	-	ref	-	-
Knowledge (acceptable) and practice (unacceptable)	27	50.9	26	49.1	53	5.2	1.3-20.1	0.02	5.8	1.4–24.0	0.02
Knowledge (unacceptable) and practice (acceptable)	19	27.5	50	72.5	69	1.9	0.5–7.3	0.35	1.9	0.5–7.9	0.4
Knowledge and practice of UPs both acceptable	51	31.9	109	68.1	160	2.3	0.6-8.4	0.19	2.3	0.6–8.9	0.2

Ref, reference; OR, odds ratio; CI, confidence interval; UPs, universal precautions.

## Discussion

This case–control study of HCWs who had sustained an NSI compared with those HCWs who had not experienced an NSI revealed important findings of HCW knowledge and practice of UPs in HCFs in the Ugu north sub-district of KZN. Both case and control HCWs achieved scores above 80% for knowledge of UPs, which is in accordance with studies conducted in other countries on HCWs’ knowledge of UPs.^7,9,12,21^

Importantly, in this study, there were HCWs among the cases (18%) and controls (29.5%) who felt that needles should be recapped after use, reflecting a gap in knowledge of UPs. Recapping of used needles is one of the main causes of NSI among HCWs.^22,23^ A systematic review of factors associated with NSIs in health care occupations in 2016 reported that recapping of needles together with instrument preparation and administering injections were responsible for the highest incidence of NSIs.^23^

Case HCWs had significantly better knowledge about the concept of UPs, potential paths of exposure for occupational infections and the frequency of hand washing which is required, when compared with controls. This better knowledge may be because HCWs who had sustained an NSI may have been made aware post-injury about the importance of UPs. However, the nature of our study design did not allow us to draw a conclusion on this outcome.

Of significance in this study is that although the case HCWs had better knowledge of UPs, the practice of UPs was significantly better among the control HCWs. This was reflected in the overall better practice score and the significantly better use of gloves (*p* = 0.03) and mask and eye protection (*p* = 0.01). Further, the logistic regression analysis showed that HCWs with acceptable knowledge and unacceptable practice of UPs had a far greater odds of having sustained an NSI (OR: 5.8), suggesting that the knowledge which case HCWs had did not translate into practice. This is similar to findings in other studies where HCWs appear to have knowledge of UPs, but it did not transfer into practice. In a tertiary hospital in Nigeria, Adegboye et al.^7^ found that although 86% of HCWs knew that infections can be transmitted by hand, only 32.5% of HCWs practised the six steps of the hand-washing technique as recommended by the WHO. In a study on nurse’s knowledge attitudes and practices conducted in two tertiary hospitals in Nigeria, researchers reported that HCWs had a median knowledge score above 90%, but the median practice score was 50.8%.^9^ The burden of evidence from the literature confirms that knowledge of UPs does not translate into practice, as reflected in our study population.

## Study limitations

Despite the important findings of this study, there were limitations in the study which need to be borne in mind. This study was limited to only one sub-district; it therefore does not indicate the knowledge and practice of UPs in the rest of the district and other districts of KZN. All of the available HCWs did not participate in this study which influenced the data by limiting the number of cases to controls. Some of the data collected may be under-reported because of recall bias, as HCWs were not always able to remember if they always practised UPs. However, in some instances, HCWs may have reported conforming to UPs because they are aware of what should be performed, even if they were not implementing correct practices, which could have resulted in an over-reporting of UP practices. Further, the level of adherence to UPs may have been better assessed by observation, although observation would have influenced the participant’s normal routines. Lastly, the researcher tried to collect the data over a short space of time, and staff may have answered differently if they had more time to reflect.

## Conclusion and recommendations

Based on the findings of this study, we conclude that although HCWs in Ugu north sub-district, KZN, are informed about UPs, implementation of their knowledge into practice is poor. In particular, there was limited knowledge with respect to recapping of needles, and practising better UPs protected HCWs from experiencing NSIs.

The better UP knowledge among case HCWs may have been influenced by their prior experience. This would be best explored further through a cohort study which would allow a baseline knowledge assessment and then a follow-up assessment after any NSI of participating HCWs.

The incorrect perception among HCWs with respect to recapping of needles in this sub district should be addressed during training on UPs. In the light of the fact that practice of UPs among HCWs is poor, it is recommended that when training about UPs, emphasis is placed on the translation of knowledge into practice. Ara et al., by using a multimodal intervention, which included monitoring and feedback of UP practices to nurses in addition to theoretical teaching, found significant improvements in the use of gloves and reductions in NSIs among nurses in five hospitals in Bangladesh.^24^ This gives credence to the recommendation that direct observation and correction of practice during in-service training sessions of HCWs should be added when training HCWs on UPs and should be reinforced in the South African setting.
